# Neurocognitive Functioning among Children with Sickle Cell Anemia Attending SCA Clinic at MNH, Dar es Salaam, Tanzania

**DOI:** 10.1155/2020/3636547

**Published:** 2020-09-01

**Authors:** Limi O. Matondo, Edward Kija, Karim P. Manji

**Affiliations:** Department of Paediatrics and Child Health, Muhimbili University, Dar Es Salaam, Tanzania

## Abstract

**Background:**

Children with sickle cell anemia are at a higher risk of developing neurological sequelae like abnormal intellectual functioning, poor academic performance, abnormal fine motor functioning, and attentional deficits. There is a paucity of data about neurocognitive impairment among children with sickle cell anemia in Tanzania. Recognition of the magnitude of neurocognitive impairment will help to provide insight in the causative as well as preventive aspects of the same. Therefore, this study was carried out to determine the prevalence and factors associated with neurocognitive impairment in children with sickle cell anemia.

**Methods:**

This is a cross-sectional comparative study between children with SCA and a control group of the hemoglobin AA sibling. It was carried out in Muhimbili National Hospital during a five-month period. The Rey–Osterrieth Complex Figure test (ROCF) which is used to test memory and visual special functions and KOH block design tools that have been previously validated through another study locally were used. Additional information on demographic characteristics was also collected using a predetermined questionnaire. Proportions and comparisons of means were used to examine associations between neurocognitive impairment and independent variables for associated factors.

**Results:**

A total of 313 children were included in the final analysis. Among all the participants, the majority of the participants in the sickle cell group were of the age group 14-15 years (45.9%). In the comparison group, the majority were of the age group 9-10 years (43.8%). The neurocognitive scores in children with sickle cell anemia were significantly different from the normal siblings. In the copy ROCF, the neurocognitive function in SCA participants was 68.2% below the mean as compared to 45% of their counterparts, *p* ≤ 0.001. Additionally, there was no difference in memory in children with SCA compared to normal siblings (14.8% vs. 12.5%, respectively, *p*=0.606). Children with SCA had a higher proportion of impaired IQ (85.4%) as compared to children without SCA (72.5%), and the difference was statistically significant, *p*=0.009. Factors associated with neurocognitive impairment were age above 13 years, BMI, and absenteeism from school. *Conclusion and Recommendation*. Children with SCA had more impairment in terms of copying and IQ. We recommend assessment at the younger age group, increased sample size in future studies, and long-term cohort follow-up.

## 1. Background

Around 5.2% of the world population carry the sickle cell hemoglobin variant [1], with sickle cell anemia (SCA) being the most clinically important. It is estimated that in Africa alone 200,000 new children are born with SCA every year [2]. The national prevalence in Tanzania in unknown, but estimates from Kilimanjaro suggest a rate of 4% [3].

One of the devastating complications of SCA is cerebral vascular accident (CVA) that affects 10% of the children with sickle cell anemia (SCA). Abnormalities on brain magnetic resonance imaging (MRI) compatible with ischemic injury were found in 23% of the children without focal neurological findings. These “silent infarcts” predispose children to neuropsychological dysfunction, as well as clinical stroke [4]. Neurological insults resulting from SCA may present with neurocognitive impairment early in life even with the evidence of normal neuroimaging [5].

Sickle cell anemia (SCA) is an inherited blood disorder characterized by abnormal red blood cells that assume a sickled shape in areas with low oxygen tension [6]. The sickled blood cells are rigid in nature and hence lose the flexibility to flow through smaller vessels leading to blockade and infarcts.

Neurocognitive functioning encompasses reasoning, memory, attention, and language and can lead directly to the attainment of information, and thus knowledge. Neurocognitive impairment is defined by difficulties in remembering, concentrating, learning, or decision-making that affect everyday life [7]. There is growing evidence that children with sickle cell anemia are likely to be cognitively impaired as compared to healthy children [8].

Mechanisms leading to neurocognitive impairment in SCA children include chronic hypoxic damage to the brain; decreased pulmonary function; recurrent microinfarction of the central nervous system (CNS); chronic nutritional deficiency associated with increased metabolic demands. The accumulative effect of this process is likely to result in compromised neurocognitive function [9]. Chronic anemia, a common feature among patients with SCA, can cause cognitive deficits indirectly by induced fatigue. It has been postulated that failure of adaptation to hypoxic events is responsible for this impairment. [10, 11]. Attention and executive functions are the most affected by this hypoxic phenomenon.

Furthermore, the developing brain in children is prone to sustain more injuries which might be irreversible. Chronic illness, such as SCA, has been associated with abnormal brain development in children with repeated episodes of injury: some of which are irreversible with loss of previously gained cognitive functioning [12]. The diagnosis of neurocognitive function can be invisible due to failure of routine imaging and diagnostic tests to detect it [13], thus children will be picked with severe deficits in function.

With developed SCA clinics like in Dar es Salaam, it should be possible to diagnose these children earlier when some remedial actions can be executed. Lifelong follow-up should help detect neurocognitive impairments and allow children with SCA to grow up as intellectual adults like anyone else. Knowing the incidence is thus important so as to understand the significance of the burden. Additional efforts to assess neurocognitive function including imaging costs are to be borne.

## 2. Methods

### 2.1. Study Area

This study was conducted at Muhimbili National Hospital (MNH), a university teaching hospital located in the commercial capital of Dar es Salaam. MNH is the first hospital in the country of over 50 million inhabitants to establish a well-organized SCA clinic with a well-established cohort of patients for follow-up and research. This clinic runs once a week with an expanded pediatric age of up to 18 years. This makes it a conducive site for the study as all study participants can be recruited from the same clinic. The clinic receives on average between 30 and 40 children per day for various reasons. Acutely sick children are admitted to the general pediatric wards as well.

### 2.2. Study Population

The study targeted all children who were attending the sickle cell clinic and were 9–15 years old. This group was used because the neurocognitive tests have been validated in this age group. They are old enough to be compliant with the study protocol, and they are more independent than the younger age.

### 2.3. Tools Used

The test to be performed (ROCF and KOH's block design) are more applicable in children between 6 and 9 years of age. These tests can be used to test executive function and visual special ability and memory.

### 2.4. Study Design

This was a health facility-based, cross-sectional descriptive study to determine prevalence of neurocognitive impairment among 9–15-year-old children with sickle cell anemia attending the MNH sickle cell clinic compared to their normal siblings. It included 233 children with SCA and 80 siblings. This study was conducted between September 2016 and February 2017.

### 2.5. Inclusion Criteria

- Children confirmed with SCA by HB electrophoresis documented in the special SCA clinic card held by each patient
- Age of between 9 and 15 years
- Siblings who were hemoglobin AA
- Children whose parents or custodians gave informed consent to participate

### 2.6. Exclusion Criteria

- All children with SCA and other chronic illness unrelated to the SCA: congenital heart disease and epilepsy

### 2.7. Sample Size Determination

The sample size was calculated by using the Kish Leslie formula, and in the calculation of sample size, the proportion used for children with SCA was 15% [14] and proportion of the siblings used was 50% [15], so the total sample size was 313 children.

### 2.8. Sampling Technique

All eligible children whose parents provided informed consent were recruited conveniently by recruiting the children who attended first in the study sample until the required sample size was attained. Recruitment of the participants was carried out exclusively on Thursdays because of the SCA clinic day.

### 2.9. Data Collection

#### 2.9.1. Recruitment

Cases were identified a day before attending the clinic from the clinic register. Records were searched to identify sick children with siblings who had tested negative for sickle cell disease and anemia. Parent/custodian telephone number in the records was then used to obtain verbal consent and were requested to bring the sick child along with the negative sibling.

#### 2.9.2. Tools

ROCF and KOH's block design were used in this study. Both these have been validated through an ongoing study at MUHAS (not yet published). The child is given an ROCF stimulus card. Once the copy was completed, the stimulus figure and the examinee's copy were removed from the view. They were then kept busy by giving them other tests within the clinic. After 15 minutes, they were called and given a white paper to draw the previously drawn diagram without seeing it (from memory: they were not previously told that they would have to redraw it), time was not to exceed 30 minutes. To test the IQ, a child was subjected to the KOH block test whereby she/he was shown 17 cards with different colored designs and asked to arrange the blocks according to the cards. The performance was based not just on the accuracy of the arrangement but also on the examiner's observation of the child's behavior during the test, including factors like attention level and self-criticism. A child's score was obtained in terms of mental age, and then the mental age was calculated in terms of percentage from the actual age of the child. A score of 80% and above was considered normal IQ.

A standardized structured questionnaire was used to collect sociodemographic information of the child and parents as well as ROCF and KOH's test results.

### 2.10. Data Analysis

Case record forms were edited for consistency and checked for quality. Data analysis was done using Statistical Package for Social Science (SPSS) version 21. Cross-tabulations were used to examine associations between neurocognitive scores indicating impairment and independent variables for associated factors.

The mean scores were considered normal neurocognitive functioning, and the interquartile scores indicated the level of neurocognitive impairment. Hence, if the child was in the lowest interquartile range, he/she was considered abnormal.

Impaired neurocognitive functioning was defined as the score below the mean for that age group among the normal siblings. Therefore, normal siblings were used as a standard. The proportion of the children with a score below the mean among the normal children in the two groups was compared.

Continuous variables were expressed as the mean, median, and interquartile range. Differences in proportions were tested using the chi-square test and Fisher's exact test where applicable. Odds ratio (OR) and 95% confidence intervals (CI) were calculated to determine the associations between dependent variables and independent variables. A *p* value of less than 0.05 was considered statistically significant. Moreover, those baseline characteristics with a *p* value less than 0.05 were analyzed by logistic regression to identify the association of independent factors with neurocognitive impairment.

### 2.11. Ethical Issues and Clearance

Ethical clearance and permission to conduct this study were sought from the MUHAS Ethical Committee and MNH Administration, respectively, with a MUHAS letter of approval with reference numbers MU/PGS/SAEC/Vol. XVI and HD/MUH/T.108/2014. Children who were found to have other medical conditions were referred to appropriate clinics accordingly to get treatment. Children who had neurocognitive impairment were counseled on various ways to improve their cognitive functioning.

## 3. Results

A total of 233 children with SCA were studied along with 80 siblings without the disease. Most of the study participants were between 14 and 15 years of age (124 (40%)) within the SCA group, while within the siblings, the majority were between 9 and 10 years. Females were the majority in both groups, with most children in both groups having a primary level of education. Proportionately, more children in the disease group had missed school compared to the siblings (Table 1).

### 3.1. Neurocognitive Functioning in the Study Population

**Figure 1:**
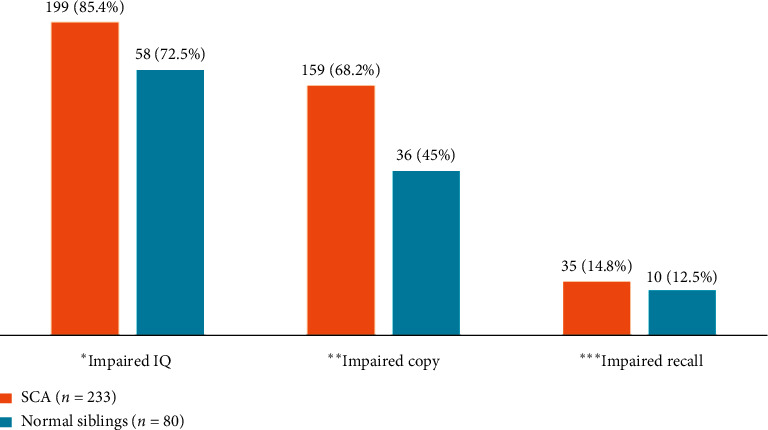
Distribution of neurocognitive impairment among SCA children when compared to their normal siblings (*n* = 313). ^*∗*^Chi-square = 6.75, *p*=0.009, ^*∗∗*^chi-square = 13.6, *p* < 0.001, and ^*∗∗∗*^chi-square = 0.26, *p*=0.606.

As can be seen in Figure 1, impairment in IQ was noted among 85.4% of the SCA group versus 72.5% in siblings, and the difference was significant (*p* 0.009). Similarly, impaired copy was significantly more among the cases compared to siblings, 68.2% and 45%, respectively (*p* 0.001). No significant difference was noted on the recall ability.

Table 2 shows the factors for impaired copy where age between 14 and 15, underweight, and school absence were the only factors that show significant association while the rest did not.

Table 3 shows the logistic regression on factors for impaired copy. The odds of developing impaired copy among SCA children were almost seven times between the age 14 and 15 years, 2.5 times for underweight, and 3.3 times for missing school more than once.

Table 4 shows the factors for impaired recall among children with SCA. Decreasing level of fathers' education and school absence were significantly associated with low recall among SCA children.

Table 5 shows the factors for impaired recall among SCA children. Primary level of education among fathers was not found to carry any increased risk of low recall.

Table 6 shows the distribution of factors for impaired IQ among children with SCA. Children of age between 14 and 15 years were more likely to have impaired IQ as well as missing school more than once.

Table 7 shows the logistic regression on factors for impaired IQ among children with SCA. The odds of having low IQ were 4.9 and 3.2 times for SCA children aged between 14 and 15 years and missing school more than once, respectively.

## 4. Discussion

Sickle cell anemia (SCA) is an inherited blood disorder characterized by abnormal red blood cells that assume a sickle shape. The sickled blood cells are rigid in nature and can be easily destructed and cause blockage of blood flow which leads to complications in different organs [6]. Cerebrovascular complications constitute one of the main causes of mortality for patients with SCA. They also are responsible for persistent neurological and/or neuropsychological deficits in surviving patients. Even more frequent are the “silent” infarctions, which are difficult to detect with a standard neurological examination and are associated with neuropsychological deficits [14]. Normal siblings performed better than the sickle cell patients on the cognitive functioning tests performed [16].

This study of neurocognitive functioning indicated a lower IQ and an impaired ROCF copying scores in children with SCA than their normal siblings. Children with SCA had more impairment in ROCF copying, and the proportion was 68% which was higher than that of the normal siblings, which was 45% as seen in Figure 1. The proportion of children with SCA who had impaired IQ was 85.4% which was higher than that of the normal siblings (72.5%).

There was no significant difference in ROCF recall between children with SCA and their healthy siblings. However, neurocognitive impairment was associated with age (*p* < 0.001), BMI (*p*=0.039), and school absenteeism (*p* < 0.001). These findings are consistent with a comparative study by Boivin and Giordani in Cameroon that showed no difference in neurocognition between children with SCA and their normal siblings [10]. But, their study had a small sample size (*n* = 96) and used the California verbal learning test for memory assessment which was different from our study [14]. Our study findings were not supported by a comparative study by Scantlebury et al. in Italy, which showed an impairment in the working memory among children with SCA compared to the normal siblings [17]. Such difference in findings could be attributed to the difference in sociodemographic features between the two studies, and their study [17] included or excluded patients with silent brain infarcts by using brain MRI which was not done in our study.

Our study showed significant differences in IQ impairment between children with SCA and normal children. Children with SCA had a higher impairment in IQ than normal siblings (Figure 1). These results appear to be consistent with a meta-analysis study by Schatz et al. [18]. In their study, they documented increased impairment in IQ in children with SCA compared to their normal siblings. This similarity could be due to resemblance in the study design, and the age groups used were comparable to the age category in our study. Although the findings are similar, there was a difference in neurocognitive tests used between our study and Schatz et al.'s study that used the Wechsler intelligent scale to assess IQ impairment [18].

Our study showed a positive association between age and neurocognitive impairment in children with SCA. SCA children in older age categories (11–13 and 14-15) were more likely to have impaired copy and IQ than young SCA children in the 9-10 year group (Table 7). This is consistent with findings by Boivin and Giordani and Schatz et al. who showed lower IQ in older SCA children (11–13 years) than in young SCA children, thus suggesting a decline in cognitive functioning with increase in age [14, 18]. The similarity between our study and the two studies is due to a similar study design and the use of children with comparable sociodemographic characteristics. Contrary to our study was the small number of participants in their studies [14, 18], and they used multiple neurocognitive tests which was different from our study which used two tests.

In our study, SCA children who missed school more than once in a month had a higher impairment in copy and IQ coupled with lower class performance compared to normal siblings (Table 6). This is contrary to the findings by Ogunfowora et al.'s study of children with SCA and their normal siblings in Nigeria which showed that there was no significant difference between school attendance and cognitive impairment among children with SCA as compared to their normal siblings [19]. This difference could be due to the use of a small sample size in their study (52 children with SCA and 42 healthy siblings), and they used children as young as 6 years and as old as 17 years [19].

### 4.1. Study Strengths and Limitations

This is the first study on neurocognitive functioning in children with SCA to be done in Tanzania. Neurocognitive functioning was assessed using well-defined and validated measures, providing a neurocognitive profile of children with SCA. As healthy siblings have been described as the preferable control group, we included a control group comprising healthy siblings of participating children with SCA, thus controlling for any confounding factors.

The robustness of this study was the neurocognitive tests used (ROCF and KOH block) which measured the visual memory and IQ. These tests are easy to administer and did not require the use of many trained personnel. Also, the tests required less time for a single test, were not invasive, and are less expensive. These factors increased the number of study participants who complied with the study during data collection.

The limitations to our study included few participants in the control group as some parents did not deem it necessary to involve the normal siblings in the study, thus reducing the strengths of our results. In our study, we did not exclude children with brain infarcts due to budget constraints (the exclusion of brain infarcts requires brain MRI). This could have affected the quality of our findings as SCA children with brain infarcts score poorly in all cognitive tests as compared to those without brain infarcts. Although both the SCA children and the normal siblings were between 9 and 15 years, children within one family did not have the same age. This could have introduced variations in our findings due to the age differences between the participants and their control from the same family.

## 5. Conclusion

1. The proportion of neurocognitive impairment in children with SCA was 68.2% in copying, 14.8% in memory, and 85.4% in IQ
2. Factors associated with neurocognitive impairment are age above 13, BMI, and school absenteeism

### 5.1. Recommendations

A larger study with more detailed neurocognitive tests which are validated in Tanzania needs to be conducted. Further studies are necessary to expand the participant pool, and thereby increase feasibility regarding the inclusion of age- and gender-matched healthy siblings from participating children with SCA. Also, future studies should consider the use brain MRI to exclude SCA children with brain infarcts. Cognitive rehabilitation tools are to be developed in MNH for assessing and follow-up of children with SCA.

The findings of this study have added more weight to the growing evidence that SCA has the potential adverse effect on the intellectual ability of affected children. In light of this, it is necessary to pay close attention to the school performance of children suffering from SCA, for early identification of underachievers who may need help in the form of extra coaching and/or home lessons.

## Figures and Tables

**Table 1:** Baseline characteristics.

Demography	Sickle cell group (*n* = 233)		Normal siblings (*n* = 80)		Total *N* (%)
	Frequency	%	Frequency	%	
Age (yrs)					
9 to 10	57	24.5	35	43.8	92 (29)
11 to 13	69	29.6	28	35	97 (31)
14 to 15	107	45.9	17	21.2	124 (40)

Sex					
Male	112	48	32	40	144 (46)
Female	121	52	48	60	169 (54)

Children education					
Primary	177	76	68	85	245 (78.3)
Secondary	56	24	12	15	68 (21.7)

How many days you missed school?					
Never	55	23.6	71	88.8	126 (40.3)
More than once	178	76.4	9	11.2	187 (59.7)

**Table 2:** Factors for impaired copy among studied children with SCA attending MNH (*n* = 195).

Factor for impaired copy	SCA group (*n* = 142)	Normal sibling group (*n* = 53)	Total	*X* ^2^, *p* value
Age (yrs)				9.17, **0.01**
9 to 10	45 (31.7)	29 (54.7)	74 (37.9)	
11 to 13	48 (33.8)	14 (26.4)	62 (31.8)	
14 to 15	49 (34.5)	10 (18.9)	69 (30.3)	

Sex				0.77, **0.378**
Male	69 (48.6)	22 (41.5)	91 (46.7)	
Female	73 (51.4)	31 (58.5)	104 (53.3)	

Children education				0.18, **0.94**
Primary	120 (84.5)	47 (88.7)	167 (85.6)	
Secondary	22 (15.5)	6 (11.3)	6 (14.4)	

BMI				4.25, **0.039**
Normal	26 (18.3)	17 (32.1)	43 (22.1)	
Underweight	116 (81.7)	36 (67.9)	152 (77.9)	

Father education				0.798, **0.671**
Primary	95 (66.9)	33 (62.3)	128 (65.6)	
Secondary	32 (22.5)	12 (22.6)	12 (22.6)	
University	15 (10.6)	8 (15.1)	23 (11.8)	

Mother education				0.38, **0.534**
Primary	98 (69)	39 (73.6)	137 (70.3)	
Secondary	44 (31)	14 (26.4)	58 (29.7)	

School absence				75.8, **<0.001**
Never	33 (23.2)	49 (92.5)	82 (42.1)	
More than once	109 (76.8)	4 (7.5)	113 (57.9)	

What is exam rank in class?				0.017, **0.895**
Within top 10	23 (16.2)	9 (17)	32 (16.4)	
Out of top 10	119 (83.8)	44 (83)	163 (83.6)	

**Table 3:** Logistic regression on factors for impaired copy among SCA studied children attending MNH (*n* = 195).

Factor for impaired copy	Crude OR (95% CI)	*p* value	Adjusted OR (95% CI)	*p* value
Age (yrs)				
9 to 10	1 (ref)		1 (ref)	
11 to 13	2.2 (1–4.7)	**0.04**	2.5 (0.9–7.1)	**0.081**
14 to 15	3.1 (1.3–7.2)	**0.006**	6.8 (2.2–21)	**0.01**

BMI				
Normal	1 (ref)		1 (ref)	
Underweight	2.1 (1–4.3)	**0.042**	2.6 (1.5–4.4)	**<0.001**

How many days you missed school?				
Never	1 (ref)		1 (ref)	
More than once	3.8 (1.9–11)	**<0.001**	3.3 (1.9–12.2)	**<0.001**

**Table 4:** Factors for impaired recall among studied children with SCA attending MNH (*n* = 45).

Factor for impaired recall	Sickle cell group (*n* = 15)	Normal siblings (*n* = 30)	Total	*X* ^2^, *p* value
Age (yrs)				
9 to 10	17 (56.7)	12 (80)	29 (64.4)	
11 to 13	6 (20)	1 (6.7)	7 (15.6)	
14 to 15	7 (23.3)	2 (13.3)	9 (20)	2.4, **0.288**

Sex				
Male	13 (43.3)	7 (46.7)	20 (44.4)	
Female	17 (56.7)	8 (53.3)	25 (55.6)	0.45, **0.832**

Children education				
Primary	28 (93.3)	14 (93.3)	42 (93.3)	
Secondary	2 (6.7)	1 (6.7)	3 (6.7)	^*∗*^ *p*=1

BMI				
Normal	7 (23.3)	5 (33.3)	12 (26.7)	
Underweight	23 (76.7)	10 (66.7)	33 (73.3)	0.5, **0.475**

Father education				
Primary	18 (60)	7 (46.7)	25 (55.6)	
Secondary	12 (40)	5 (33.3)	17 (37.8)	
University	—	3 (20)	3 (6.7)	6.4, **0.04**

Mother education				
Primary	22 (73.3)	10 (66.7)	32 (71.1)	
Secondary	8 (26.7)	5 (33.3)	13 (28.9)	0.21, **0.642**

School absence				
Never	2 (6.7)	15 (100)	17 (37.8)	
More than once	28 (93.3)	—	28 (62.2)	37, **<0.001**

What is your position in class?				
Within top 10	6 (20)	4 (26.7)	10 (22.2)	
Out of top 10	24 (80)	11 (73.3)	35 (77.8)	0.25, **0.612**

^*∗*^Fisher's exact test was used.

**Table 5:** Logistic regression on factors for impaired recall among SCA studied children with SCA attending MNH (*n* = 45).

Factor for impaired recall	Crude OR (95% CI)	*p* value
Father education		
Secondary	1 (ref)	
Primary	1.1 (0.3–4.2)	**0.921**

**Table 6:** Factors for impaired IQ among studied children with SCA attending MNH (*n* = 257).

Factor for impaired IQ	Sickle cell group (*n* = 194)	Normal siblings (*n* = 63)	Total	*X* ^2^, *p* value
Age (yrs)				
9 to 10	44 (22.7)	28 (44.4)	72 (28)	
11 to 13	55 (28.4)	20 (31.7)	75 (29.2)	
14 to 15	95 (49)	15 (23.8)	110 (42.8)	15.2, **<0.001**

Sex				
Male	87 (44.8)	24 (38.1)	111 (43.2)	
Female	107 (55.2)	39 (61.9)	146 (56.8)	0.88, **0.347**

Children education				
Primary	147 (75.8)	53 (84.1)	200 (77.8)	
Secondary	47 (24.2)	10 (15.9)	57 (22.2)	1.92, **0.166**

BMI				
Normal	49 (25.3)	19 (30.2)	68 (26.5)	
Underweight	145 (74.7)	44 (69.8)	189 (73.5)	0.58, **0.444**

Father education				
Primary	128 (66)	43 (68.3)	171 (66.5)	
Secondary	42 (21.6)	13 (20.6)	55 (21.4)	
University	24 (12.4)	7 (11.1)	31 (12.1)	1.22, **0.94**

Mother education				
Primary	141 (72.7)	50 (79.4)	191 (74.3)	
Secondary	53 (25.7)	13 (20.6)	66 (25.7)	1.1, **0.29**
School absence				
Never	44 (22.7)	56 (88.9)	100 (38.9)	
More than once	150 (77.3)	7 (11.1)	157 (61.1)	87, **<0.001**

What is your position in class?				
Within top 10	31 (16)	9 (14.3)	40 (15.6)	
Out of top 10	163 (84)	54 (85.7)	217 (84.4)	0.11, **0.747**

**Table 7:** Logistic regression on factors for impaired IQ among SCA studied children attending MNH (*n* = 257).

Factor for impaired IQ	Crude OR (95% CI)	*p* value	Adjusted OR (95% CI)	*p* value
Age (yrs)				
9 to 10	1 (ref)		1 (ref)	
11 to 13	1.7 (1.1–3.5)	**0.014**	2.5 (1–6.2)	**0.081**
14 to 15	4 (1.9–8.2)	**<0.001**	4.9 (2–12.4)	**<0.001**

How many days you missed school?				
Never	1 (ref)		1 (ref)	
More than once	3.9 (1.7–9)	**<0.001**	3.2 (2.5–15.4)	**<0.001**

## Data Availability

The data used to support the findings of this study are included within the article.
